# Lesion quantification and detection in myocardial ^18^F-FDG PET using edge-preserving priors and anatomical information from CT and MRI: a simulation study

**DOI:** 10.1186/s40658-016-0145-4

**Published:** 2016-06-17

**Authors:** Anna Turco, Johan Nuyts, Olivier Gheysens, Jürgen Duchenne, Jens-Uwe Voigt, Piet Claus, Kathleen Vunckx

**Affiliations:** KU Leuven - University of Leuven, Department of Imaging and Pathology, Nuclear Medicine and Molecular Imaging, Medical Imaging Research Center (MIRC), Herestraat 49, Leuven, 3000 Belgium; University Hospitals Leuven, Department of Nuclear Medicine, Herestraat 49, Leuven, 3000 Belgium; KU Leuven - University of Leuven, Department of Cardiovascular Sciences, Cardiology, Medical Imaging Research Center (MIRC), Herestraat 49, Leuven, 3000 Belgium; University Hospitals Leuven, Department of Cardiovascular Diseases, Herestraat 493000, Leuven, Belgium

**Keywords:** MAP reconstruction, Cardiac ^18^F-FDG PET, Anatomical priors, Edge-preserving priors

## Abstract

**Background:**

The limited spatial resolution of the clinical PET scanners results in image blurring and does not allow for accurate quantification of very thin or small structures (known as *partial volume effect*). In cardiac imaging, clinically relevant questions, e.g. to accurately define the extent or the residual metabolic activity of scarred myocardial tissue, could benefit from partial volume correction (PVC) techniques.

The use of high-resolution anatomical information for improved reconstruction of the PET datasets has been successfully applied in other anatomical regions. However, several concerns linked to the use of any kind of anatomical information for PVC on *cardiac* datasets arise. The moving nature of the heart, coupled with the possibly non-simultaneous acquisition of the anatomical and the activity datasets, is likely to introduce discrepancies between the PET and the anatomical image, that in turn might mislead lesion quantification and detection. Non-anatomical (edge-preserving) priors could represent a viable alternative for PVC in this case.

In this work, we investigate and compare the regularizing effect of different anatomical and non-anatomical priors applied during maximum-a-posteriori (MAP) reconstruction of cardiac PET datasets. The focus of this paper is on accurate quantification and lesion detection in myocardial ^18^F-FDG PET.

**Methods:**

Simulated datasets, obtained with the XCAT software, are reconstructed with different algorithms and are quantitatively analysed.

**Results:**

The results of this simulation study show a superiority of the anatomical prior when an ideal, perfectly matching anatomy is used. The anatomical information must clearly differentiate between normal and scarred myocardial tissue for the PVC to be successful. In case of mismatched or missing anatomical information, the quality of the anatomy-based MAP reconstructions decreases, affecting both overall image quality and lesion quantification. The edge-preserving priors produce reconstructions with good noise properties and recovery of activity, with the advantage of not relying on an external, additional scan for anatomy.

**Conclusions:**

The performance of edge-preserving priors is acceptable but inferior to those of a well-applied anatomical prior that differentiates between lesion and normal tissue, in the detection and quantification of a lesion in the reconstructed images. When considering bull’s eye plots, all of the tested MAP algorithms produced comparable results.

## Background

Positron emission tomography (PET) images suffer from partial volume effects (PVE) due to the limited spatial resolution of the PET system (2–6 mm FWHM [1, 2]). This effect is not only observed in the heart, but all anatomies can be affected. However, there are very thin or small structures in the heart (e.g. apex, right ventricle) that can be particularly affected by PVE. Moreover, some pathologies might cause a thinning or scarring of the myocardial walls. In all these cases, the blurring caused by PVE might hamper correct interpretation of the resulting image. In order to deal with the PVE in PET, many partial volume correction (PVC) techniques have been proposed [3].

In cardiac imaging, additional blurring is caused by the breathing motion and the beating of the heart. Dual gating of the cardiac datasets is one possible approach to remove the motion blur [4]. This approach removes the motion blur that affects the PET acquisitions by dividing the initial PET list-mode into a set of *gates*, each of which ideally represents the heart in a fixed cardiac and respiratory phase. Despite being effective in removing the motion blur in most cases, the process of gating dramatically reduces the statistics of the final datasets and leads to extremely noisy reconstructions (still to be corrected for PVEs). Alternatively, the use of motion fields extracted e.g. from the PET dataset itself [5, 6] or from a simultaneous dynamic magnetic resonance imaging (MRI) scan [7] would correct for the motion and improve the noise characteristics of the final PET dataset, but the PVE would still need to be corrected for.

Among the methods that have been proposed in the past to tackle the PVEs, an effective way is represented by the incorporation of the resolution effects into the system matrix during the iterative image reconstruction process (resolution recovery (RR)). This process can effectively deal with PVEs but, due to the ill-posed nature of the problem, reconstruction with resolution recovery can lead to over- and under-shoots of the reconstructed activity, known as Gibbs artefacts, that might hamper accurate image quantification [8]. Consequently, some sort of regularization is needed to avoid such artefacts. The use of high-resolution anatomical information (e.g. high-resolution computed tomography (HRCT) or MRI) for improved reconstruction of the activity datasets is appealing and has shown promising results in brain imaging [9]. Clinically relevant questions in the field of cardiac ^18^F-FDG PET, e.g. to accurately define the amount of tissue still metabolically active in or around a lesioned site, could benefit from anatomy-based PVC. The acquisition of the anatomical image for PVC is, however, not free from drawbacks. Firstly, the entire examination would be more expensive, longer and more cumbersome than a single PET study. In fact, most PET scanners are hybrid devices that include a CT module, with which a CT for attenuation correction can be acquired. However, the spatial and temporal resolution of most CT modules in the current PET/CT scanners is often insufficient for obtaining a frozen image of the heart in one cardiac and respiratory phase, and with a quality that is adequate for PVC. Therefore, the patient would need to be transferred from the PET scanner to a dedicated scanner for the acquisition of the image to be used as anatomical information. The use of truly simultaneous PET/MR devices would overcome this issue. This technology, however, is currently being integrated in the clinical practice, and so far, very few centres can benefit from its advantages.

Moreover, discrepancies between the selected PET gate and the anatomical gate being used are likely to occur, especially if the two acquisitions come from scanners of two different vendors. In addition, small, residual motion artefacts might still be present after the gating of both datasets, due to changes in the heartbeat or breathing patterns from one scan to the other. Furthermore, the high level of noise present in the PET images, coupled with possible residual motion artefacts and attenuation artefacts [10], might complicate the perfect alignment of the two datasets and therefore increase the chances to introduce additional, anatomy-driven artefacts in the PVC-PET reconstructions. The reconstruction of the PET datasets with the use of anatomical prior information is also not straightforward and more time consuming, as it needs a case-by-case verification of the alignment between the anatomy and the activity images and, if necessary, extra steps (e.g. non-rigid registration or manual initialization of the alignment of the datasets) in order to obtain an acceptable alignment before the actual reconstruction can take place.

On the other hand, edge-preserving and de-noising techniques, which promise noise reduction and edge preservation without the use of any anatomical side information, have also been presented in the past to deal with the Gibbs artefacts caused by the RR [11, 12]. These techniques produce visually appealing images, with better contrast-to-noise ratios when compared to the current standard for PET reconstruction. In addition, they have the advantage of not relying on an external, additional scan for anatomy. Therefore, they are not subject to the previously mentioned complications and they could more easily be introduced in the clinical practice and in the clinical software.

The aim of this article is threefold. Firstly, we aim at assessing the performances of edge-preserving priors for the purpose of lesion detection in cardiac ^18^F-FDG PET, in comparison to anatomy-based priors. Secondly, we aim at highlighting the possible risks of anatomy-based PVC, which occur when the anatomical image is mismatched or shifted relative to the corresponding PET dataset, and their effect on lesion detection and quantification. Finally, we aim at identifying differences in performance when using two different modalities as source of anatomical information. In particular, we compare the use of an MR image where the lesion is visible, to the use of a high-resolution CT image where the lesion is not visible.

This study explores the effect of PVC on lesion visibility and quantification, in the ideal-case scenario where all motion has been already removed from the datasets. In fact, only in this way can the blurring caused by the PV effect be clearly distinguished from the one caused by the motion. In doing so, we can therefore clearly identify how well the sole blurring due to PVE is eliminated thanks to edge-preserving and anatomical priors. XCAT-based simulations have been performed to achieve the aforementioned objectives.

## Methods

### Phantom generation

The XCAT software [13] was used to create the ground truth activity distribution, the attenuation distribution and the high-resolution anatomical images to be used for this study. We generated an XCAT phantom corresponding to a single cardiac and respiratory gate for the PET, the attenuation and the high-resolution anatomical images.

**Fig. 1:**
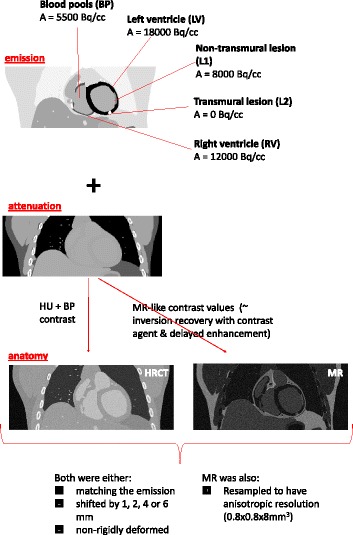
Overview of the activity, attenuation and anatomical datasets generated with the XCAT phantom. The anatomical images are obtained starting from the attenuation image generated by the XCAT software and post-processed manually to obtain the desired anatomical information

All phantoms represented an average male, arms up [14]. Realistic and homogeneous activity values were assigned to the different tissues of the simulated activity phantom (see Table 1), based on average activity values observed in available measured datasets (normally injected with 370 MBq and scanned 30 min postinjection). Values in the same order of magnitude were also reported upon in a previous study on myocardial ^18^F-FDG uptake [15]. An overview of the main common parameters used to generate all the phantoms can be found in Table 1. Figure 1 shows a coronal slice of the generated datasets.

**Table 1:** Key parameters for phantom generation

Antero-posterior (AP) expansion [cm]	1.2
Diaphragm motion [cm]	2.0
Resp. cycle duration [s]	5
Card. cycle duration [s]	1
No. resp gates/cycle	5
No. card gates/cycle	10
Phantom size [pixel]	600 × 600 × 203
Phantom pixel size [mm]	0.8
Left ventricle activity [kBq/cc]	18
Right ventricle activity [kBq/cc]	12
Lung activity [kBq/cc]	0.9
Blood pool activity [kBq/cc]	5.5
Liver activity [kBq/cc]	6.5
Non-transmural lesion (L1) activity [kBq/cc]	8
Transmural lesion (L2) activity [kBq/cc]	0

#### PET and AC CT datasets

One static image of the thorax was obtained with the XCAT software, with the heart captured in a fixed cardiac and respiratory phase. The gate corresponding to end-diastole was chosen. The respiration phase of the simulated PET phantom was kept fixed to end-expiration. This procedure simulates a cardiac ^18^F-FDG PET scan where the cardiac and respiratory gating have ideally removed all motion present in the dataset. Two lesions (transmural and non-transmural) were included in the simulation. The non-transmural lesion (L1) transgresses 60 % of the mid lateral wall and has a volume of approximately 3 ml. The transmural lesion (L2) is located in the apical portion of the inferior wall and has a volume of 1.5 ml (Fig. 1).

A corresponding attenuation image at 511 keV was automatically generated with the XCAT software, in the same cardiac and respiratory phase as the PET, used to perform attenuation simulation and attenuation correction (AC) of the PET dataset. Both the attenuation and the activity images were created using a voxel size of 0.8 mm in all directions.

#### Anatomical images (HRCT and MRI)

In order to generate the HRCT frame of reference, a dataset with blood-pool contrast and high spatial and temporal resolution was generated. To this purpose, the AC CT described above was converted to Hounsfield units (HU) and blood contrast enhancement was performed by thresholding the blood from resulting images. This resulted in a simulated high-resolution contrast-enhanced CT image to be used as perfectly matching anatomical information during the reconstruction of the PET dataset.

A second anatomical image was also obtained by shifting such perfectly matching anatomical image by 2 mm both in the x and in the z direction (the corresponding reconstruction has the suffix *-shift*), with the aim of verifying the robustness of the anatomy-based prior to mis-registration. In addition, to verify the robustness of the conclusions against varying magnitudes and directions of shifts of the anatomical image relative to the PET, ten random directions were generated in 3D and used to obtain shift vectors with a magnitude of 1, 2, 4, and 6 mm (40 vectors in total). These shift vectors were each applied to the perfectly matching anatomical image to offset it from the activity image.

**Fig. 2:**
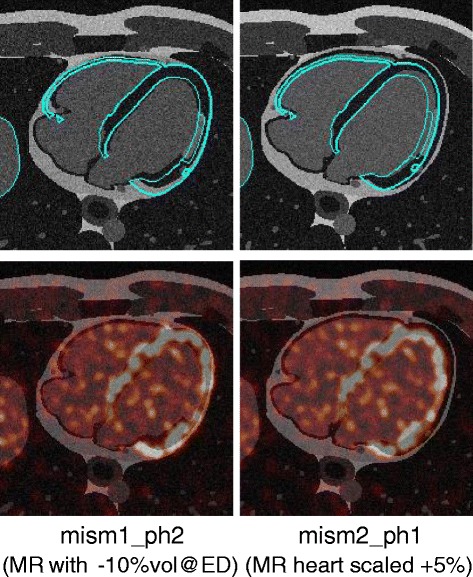
Mis-alignment of PET and MR datasets. Two examples of mismatched MRs: on the *left*, the diastolic volume in the MR is reduced by 10 % and the next cardiac phase is chosen relative to the PET. On the *right column*, the heart of the MR is up-scaled by 5 % when compared to the heart of the corresponding PET dataset. On the *top row*, the contours of the initial activity phantom are overlapped in *blue*. In the *bottom row*, the same phantom was reconstructed using OSEM3D with RR and post-smoothing of 5 mm (*red colour scale*) and it was overlapped to the MR images (*grey colour scale*). While in the *top row*, the misalignment between PET and MR is very easily detectable, it would be harder to identify when using a realistic PET image

Another anatomical image was generated in the same respiratory phase and in a slightly different cardiac phase from the activity image (the corresponding reconstruction are referred to with the suffix*-mism*). Four other mismatched anatomical images were simulated too, in order to account for physiological variations of the cardiac volume between different acquisitions [16] or between different heart cycles [17, 18]. To this end, we generated an anatomical image with a diastolic volume reduced by 10 % when compared to the PET image (*mism1*). Moreover, we simulated an increase of the heart size by 5 % (*mism2*), to account for small increments of the diastolic volume. The same cardiac phase (*ph1*) or the next cardiac phase (*ph2*)—compared to the PET dataset—were used for PVC. The mismatches produced were visually compared to the ground truth and were found representative of a realistic scenario (see Fig. 2).

It is important to highlight that an HRCT cannot distinguish between healthy tissue and scar. A previous preliminary study has suggested that this lack of differentiation might hamper the performances of the anatomical priors used during reconstruction of the PET datasets [19]. Conversely, other imaging modalities (e.g. MRI) are able to produce images where a clear distinction between scarred and normal tissue is present, if particular acquisition sequences are used [20]. Therefore, all anatomical images generated above (perfectly matching, shifted and in a different cardiac phase or volume) were simulated with MR-like contrast values, and also used as anatomical information during the reconstruction of the PET datasets.

**Fig. 3:**
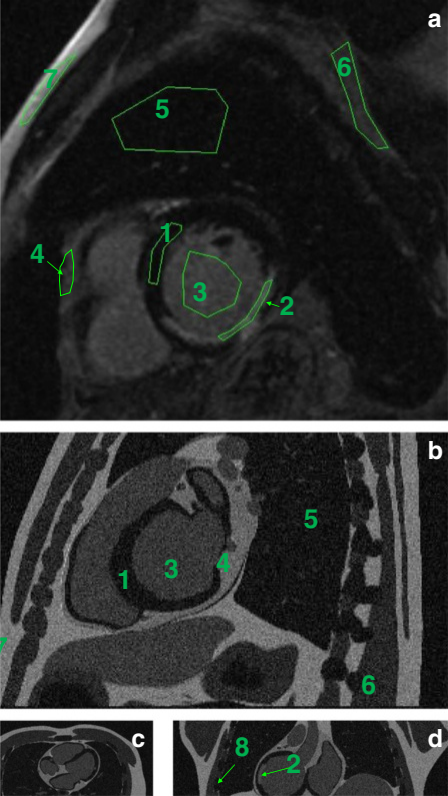
Assignment of tissue values. ROIs were delineated in a clinical MR (**a**). Similar contrast values were imposed to the tissues in the attenuation image generated with the XCAT phantom. Panes **b**, **c** and **d** represent the sagittal, transaxial and axial views of the XCAT phantom, respectively, after conversion to MR-like contrast values

The MR image was derived from the XCAT attenuation image by assigning MR-like values to the tissues. These values were extrapolated from one MR patient scan in short-axis orientation, acquired with inversion recovery and contrast enhancement. Regions of interest were drawn in seven tissue classes (left ventricle, blood pools, cardiac lesion, pericardium, lungs, soft tissue and fat), and the contrast between the different tissues was calculated. These same contrasts were reproduced in the simulated MR image, by assigning a value of 100 to the fat tissue and setting the values for the other tissue classes accordingly. The pericardium was separately generated and integrated into the just described simulated MR, as in the regular AC CT, no distinction between pericardium and muscle is possible. The bone tissue (present in the AC CT simulated dataset) was assigned a value of 0 in the simulated MR image. Rician noise was also added to the noise-free XCAT-MR according to [21]. Figure 3 shows the regions drawn in the MR and the resulting synthetic, XCAT-based MR used as anatomical prior. Table 2 shows the tissue classes and the values used for each of them, in one patient dataset and in the simulated dataset. Comparable contrast values were observed in other two MR human datasets available, acquired with a similar protocol.

**Table 2:** Simulated vs real MR values

ROI no.	Tissue	MR intensity	XCAT
		[mean ± sd in ROI]	values
1	Left ventricle (LV)	8 ± 2	10
2	Lesion	43 ± 6	55
3	Blood	35 ± 3	45
4	Pericardium	50 ± 5	70
5	Lung	8 ± 2	10
6	Soft tissue	24 ± 3	30
7	Fat	78 ± 12	100
8	Bone	n.a.	0

Furthermore, we simulated the more realistic case where the MR has an anisotropic resolution (i.e. the slice thickness is much larger—8 mm—than the in-plane resolution—0.8 mm). The reconstructions obtained with the use of such anisotropic MR are referred to with the suffix *-aniso* in the remainder of this study.

### Projection of the activity phantom

The activity distribution generated with the XCAT software and corresponding to the PET gate of interest was forward projected with a ray-tracing projection method using in-house developed software that simulates an acquisition with the PET component of a Siemens Biograph 16 PET/CT (Hirez) scanner [2]. Attenuation and a shift-invariant camera resolution were modelled, but scatter and randoms were not. The camera resolution was modelled as a Gaussian convolution (FWHM = 4.3 and 4.5 mm in the transaxial and axial direction, respectively). As for attenuation, a sinogram containing the attenuation correction factors was computed from the forward projection of the image containing the PET attenuation coefficients, generated by the XCAT software, with a ray-tracing projection method.

A noise-free sinogram was obtained. In addition, 25 noisy sinograms were generated by corrupting the noise-free sinogram with Poisson noise, corresponding to a 36-s acquisition. The scan time of 36 s corresponds to a single gate taken from a doubly gated 30 min PET scan, using five respiratory and ten cardiac gates. The simulated total number of counts amounts to 3.9 million, which is in good agreement with what was observed in previously performed animal and human thorax studies with similar activity concentrations.

We also simulated the (non-ideal) case where the respiratory motion is not corrected for. Since, in the clinical practice, the tracking of the breathing motion is much less common than the tracking of the heart rhythm with an ECG machine, we simulated the case where the *respiratory* motion is *not corrected* and only the cardiac motion is. To do so, we generated a phantom with 5 × more counts by combining the data from all breathing phases. This corresponds to taking a single cardiac gate from an ECG-gated PET scan lasting 30 min using ten cardiac gates, for an equivalent scan duration of 180 s (instead of 36 s). In addition, we simulated the case where the respiratory motion is ideally removed from the initial list-mode dataset (e.g. by list-mode motion compensation of the respiratory motion, for example as in [22]), and ECG gating is performed on top of this. As in the previous case, the equivalent cardiac frame duration would be 180 s, but this case would result in sharp images as the motion blur due to the respiration has been eliminated.

### Reconstruction of the activity phantom

The activity sinogram was reconstructed using several reconstruction algorithms, listed below. In-house developed software was used to perform reconstruction with a ray-tracing projection method. The voxel size of the reconstructed PET images was set to 1.35^3^ mm^3^. The iteration scheme used for all reconstructions consisted of 3i42s (i, iterations; s, subsets), followed by 2i24s and 2i1s. Where Gaussian post-smoothing was performed, we used a spatially-invariant Gaussian kernel with FWHM = 5 mm in the three directions. The same attenuation sinogram as for the forward projection was used during each iteration of the reconstruction of all the datasets. This excludes the effects of inaccurate attenuation correction from this simulation study. The same reconstruction procedure was applied to all the noisy sinograms.

Several reconstruction algorithms were used to reconstruct the activity dataset:

- A 3D ordered-subsets expectation-maximization (OSEM) algorithm without resolution recovery (*OSEM3D*), with and without Gaussian post-smoothing.
- A 3D OSEM algorithm with resolution recovery (OSEM3D+RR), with and without Gaussian post-smoothing. In this work, resolution recovery was achieved by modelling the resolution of the camera with a Gaussian smoothing kernel whose FWHM corresponds to the scanner’s spatial resolution. A perfect modelling of the resolution was assumed, by using the same kernel (FWHM = 4.3/4.5 mm in the transaxial and axial directions, respectively) during the simulation and the reconstruction of the datasets. At each forward and backward projection step of each iteration of the OSEM reconstruction algorithm, the intermediate reconstructed volumes were smoothed with such convolution kernel. In this way, the resolution of the scanner is taken into account during reconstruction and a de-blurred reconstructed image can eventually be obtained. However, Gibbs artefacts might be present in the final reconstructed image.
- Two maximum-a-posteriori (MAP) reconstructions with edge-preserving priors (relative difference (RD) [23] and total variation (TV) [24]) applied during reconstruction. These priors are one simple way to encourage piecewise smoothness of the reconstructions, so that the Gibbs artefacts and the noise that affect the reconstructions with RR can be mitigated or eliminated. However, since they rely on intensity differences rather than knowing the actual image boundaries, they can smooth the activity over regions that should be considered as distinct.
- MAP reconstructions with anatomical information (HRCT, isotropic MR or anisotropic MR). The anatomical information could be perfectly matching (*-perf*), rigidly shifted (*-shift*) or mismatched (*-mism*) relative to the PET dataset. Local smoothness of the activity image was encouraged (using RD with *γ*=0) within the boundaries of homogeneous regions in the anatomical image during reconstruction, according to the technique proposed by Bowsher [25] and modified by our group [26].

The general expression for the estimation of the activity distribution $\hat {\Lambda }$ using prior information is in the form:

$$\begin{array}{*{20}l} \hat{\Lambda} = \underset{\Lambda}{\operatorname{argmax}} [ L(\Lambda) - P(\beta, \gamma, \Lambda)],  \end{array} $$

where L is the log-likelihood of the activity *Λ* and P is the penalty term, where the penalty quantifies how strongly the estimated image disagrees with the prior information. *β* is the weight of the priors, and *γ* controls the edge preservation of the RD-prior (where *γ*=0 corresponds to no perservation of edges). All reconstructions with priors were performed using several parameter sets. For the RD prior, we did reconstructions using *β*= 4, 10, 30 and 50 and *γ*= 10 and 100, whereas for the TV prior, we reconstructed using *β*= 0.0001, 0.001, 0.003, 0.005, 0.01, 0.05 and 0.1. For each anatomical Bowsher prior, we tested different weights (*β*= 0.1, 4, 10, 30 and 50) and a spherical neighbourhood of 3 × 3 × 3 voxels (18 actual neighbours, excluding the eight most distant ones to obtain an approximately spherical neighbourhood) was chosen. Moreover, a fixed number of neighbours (*n*) within the neighbourhood needs to be set, to define over how many voxels the smoothing is applied. We tested the case where *n*= 3, 6, 9 and 13.

### Choice of the reconstruction parameters

#### Bias-noise plots

As described at the end of the previous section, several parameters were tested for the MAP reconstructions, but only a few parameter sets were used for the full analysis of the lesions. The bias-noise plots and the contrast-noise plots for the LV and the non-transmural lesion (L1), respectively, were used to select the parameter sets to be used for subsequent further analysis. In other words, the parameter selection has to be seen as preparatory work for the main investigation. The Bowsher reconstructions with the MR were used to assess the bias, the contrast and the noise of the reconstructions with anatomical priors.

To generate bias-noise plots, the bias was calculated as:

$$\begin{array}{*{20}l} {bias}_{LV} & = \overline{LV}_{recon} - {LV}_{true}  \end{array} $$

The contrast was computed as:

$$\begin{array}{*{20}l} {contrast}_{L1} & = 1 - \frac{\overline{L1}}{\overline{LV}}, \\[-15pt]  \end{array} $$

and the noise was computed as:

$$\begin{array}{*{20}l} {noise}_{LV} & = \overline{stddev(LV)},  \end{array} $$

where $\overline {LV}$ and $\overline {L1}$ represent the mean activities calculated in the LV and in L1 over 20 noise realizations, respectively. $\overline {stddev(LV)}$ is obtained by calculating the standard deviation on the activity in every voxel in the LV over all noise realizations and then taking the average over all voxels in the LV. Both LV and L1 were defined by thresholding the re-sampled original phantom. All voxels whose value (after the resampling of the phantom) was at least 70 % of the original activity value of the LV (or L1) were used to create the mask for the two regions.

#### Image profiles

**Fig. 4:**
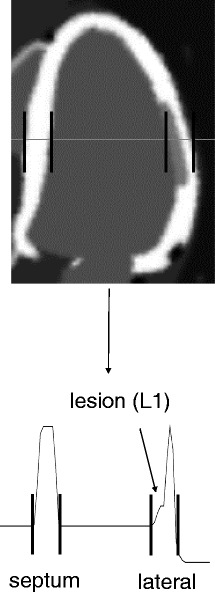
Line segment over which profiles were computed. The *bottom pane* shows the true line profile

The profiles through the middle slice of each reconstruction were also analysed, in order to verify if the parameters chosen based on the bias-noise plots could perform well enough also for what edge preservation is concerned. Figure 4 illustrates the profile, crossing over the non-transmural lesion, over which the intensity profile was computed. The profile in the septal-lateral direction was considered. The profiles of the various noise-free anatomy (MR)-enhanced images, obtained by reconstructing the same dataset with different weights and different numbers of neighbours, were plotted against the ground truth.

### Evaluation of results

The bias-noise plots and the line profiles allowed us to choose which parameter sets were the best compromise in terms of intensity preservation, noise properties of the reconstructions and ability to preserve the edges. Once the reconstructions that yielded the best compromise between these three properties were selected, the images were carefully evaluated by an experienced nuclear medicine cardiologist, who visually chose the reconstruction (among those with the best bias-noise-edge compromise) believed to be the most clinically acceptable. Once the optimal reconstruction parameters were chosen for all the selected algorithms (Bowsher, RD, TV), the analysis of the lesion areas was performed. The resulting images were evaluated against the ground truth represented by the resampled original datasets generated with the XCAT software.

#### Figures of merit

A quantitative evaluation of the reconstructions was performed. Three figures of merit were used to analyse the quantitative accuracy and give an indication of the diagnostic value of the reconstructions:

- The recovery coefficient (RC) is defined as follows:
  $$\begin{array}{*{20}l} {RC}_{r} & = \frac{\bar{r}_{recon}}{r_{true}}, \hspace{8mm} r = LV, RV, L1,  \end{array} $$
  where $\bar {r}$ represents the mean activity in the volume of interest *r*, averaged over the 25 noise realizations, and LV, RV and L1 are the volumes of interest containing the left ventricle, the right ventricle and the non-transmural lesion, respectively (see Fig. 1). The RC was not calculated for the transmural lesion L2, as its true mean value is zero.

- The contrast recovery coefficient (CRC) was used to identify the algorithm that best preserves the contrast between the region of interest *r*and the background region *b*, when compared to the ground truth contrast. The CRC was computed as in [27]:
  $$\begin{array}{*{20}l} {CRC}_{r} & = \frac{contrast_{r}^{recon}}{contrast_{r}^{true}}, r = LV, RV, L1, L2,  \end{array} $$
  where
  $$\begin{array}{@{}rcl@{}} {contrast}_{r} = \left\{ \begin{array}{ll} \frac{\bar{r}}{\overline{BP}} -1 & \text{if r = LV, RV}  \\ 1 - \frac{\bar{r}}{\overline{LV}} & \text{if r = L1, L2}.  \end{array}\right. \end{array} $$
  BP and L2 are volumes of interest containing the blood pool and the transmural lesion, respectively (see Fig. 1).

- The contrast-to-noise ratio (CNR) is another common measure for determining the performance of an algorithm. In fact, while the CRC indicates how well the intensity values of a region are recovered compared to a background region, it does not give any indication on how well the regions are visible when the noise corrupts the reconstructions. In other words, if e.g. a contrast of 10 units is observed between a lesion and the background, but the noise has a comparable magnitude, the lesion will be basically invisible despite the good contrast recovery indicated by the CRC. The CNR was defined as in [28]:
  $$\begin{array}{*{20}l} {CNR}_{r} & = \frac{contrast_{r}^{recon}}{noise_{b}^{recon}}, r = LV, RV, L1, L2,  \end{array} $$
  where
  $$\begin{array}{@{}rcl@{}} {noise}_{b} & = & \frac{\overline{stddev(b)}}{\overline{b}},  \\  b & = &\left\{ \begin{array}{ll} BP &\ \ \text{if r = LV, RV} \\  LV &\ \ \text{if r = L1, L2}. \end{array}\right. \end{array} $$

A paired *t* test was performed to identify the significant differences in CRC and CNR of the various reconstructions, compared to TV. To this end, a significance level of 0.01 was chosen for all evaluations.

#### Bull’s eye plots (polar maps)

17-segments polar maps (PMs) or bull’s eye plots of the LV were created to further evaluate the visibility and the quantification of the lesions, using a tool commonly adopted by nuclear medicine physicians. For each of the reconstructed datasets and for the ground truth, the bull’s eye plots were created as described in [29]. The delineation of the LV was automatically performed by in-house software. A fixed thickness of the LV of 6 mm was imposed, which was verified to fit the simulated LV shape well.

**Fig. 5:**
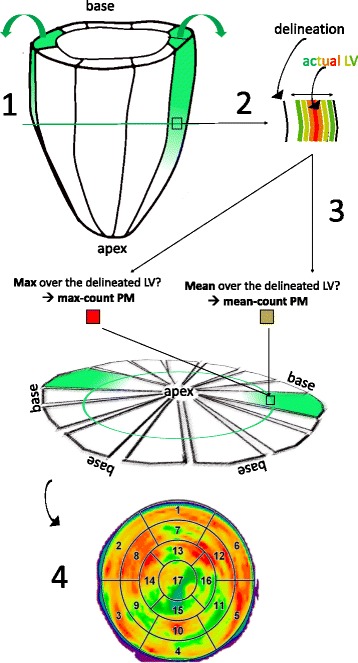
Creation of a polar map (PM) of the LV. The polar maps, or bull’s eye plots, are 2D representations of a 3D cone-shaped object (the left ventricle) (*1*). By using polar maps, we lose the information related to the distribution of activity within the wall thickness, as only one activity value can be assigned to each angular and longitudinal position of the LV (*2*). The value that is commonly assigned to each pixel of a bull’s eye plot can be the maximum, or the mean value over the LV wall at that angular and longitudinal position (*3*). Other values can be assigned, too, but the mean or the maximum along the wall thickness are the most commonly used when bull’s eye plots are considered. This assigned value is an approximation of the activity over the LV thickness. In most cases, it manages to convey an acceptable, general idea of the myocardial distribution of activity. However, in some cases, it might come in defect: for example, when overshoots of activity appear in the reconstructed images as a side effect of resolution modelling during reconstruction, or when images are extremely noisy, the max-count polar maps would assign to the PM values that are an overestimation of the actual mean activity within the LV. On the other hand, the use of mean-count polar maps might be incorrect if the delineation of the myocardial walls is not accurate (e.g. due to the blurring caused by PVE). No matter the method used to select the polar map values, the resulting image is a 2D map of the activity distribution of the LV (*4*), where the different sections (or *segments*) can be identified using different conventions. In (*4*) the “17-segment” subdivision is used, as it is the most widespread in nuclear medicine analyses (used in this work)

Each pixel in the polar map represents a small transmural portion of the LV wall, enclosed by the endocardial and epicardial contours, for each angular location and at each position along the long axis of the LV. The intensity assigned to each pixel can be computed in two ways, as illustrated in Fig. 5. On the one hand, the maximum count over the delineated LV can be taken. This method produces polar plots (*max-count polar maps*) that are less affected by delineation errors or inaccuracies, but they can reflect the noise or the artefacts introduced by the reconstruction algorithm (e.g. Gibbs over-shoots). In addition, this method will consistently take the maximum value over the thickness of the myocardium, thus possibly hiding the presence of e.g. a non-transmural lesion. Conversely, it is possible to take the mean value over the thickness of the LV, at each angular location and at each position along the long axis, and assign that mean value to the corresponding voxel in the polar map. The *mean-count polar maps* are more prone to delineation errors but, if the delineation is reliable, have the advantage of depicting more accurately the distribution of activity in the LV region considered.

Both polar map types were generated in this work. When the mean-count polar maps were computed, the delineation coming from the Bowsher reconstructions with the MR as anatomical prior information was used as delineation also for all other reconstructions. The mean value of each of the 17 segments that composed each polar map was computed, for each of the noisy reconstructions. Three regions were additionally identified in each polar map: L1 (segment 11, corresponding to the location of the non-transmural lesion), L2 (segment 13, corresponding to the location of the transmural lesion) and normal tissue (all other segments). The mean values of the normal vs lesion regions were calculated and compared to verify which algorithm best preserved the lesion contrast and best reflected the values obtained from the polar map of the ground truth.

#### Application to an animal dataset

As a proof of concept, we applied some of the techniques described in this simulation study (OSEM3D, OSEM3D+RR, TV) retroactively on a previously measured animal (sheep) dataset, where a lesion in the right ventricle and a lesion in the apical portion of the left ventricle had acutely formed before the PET scan. No Bowsher reconstructions were performed for this dataset due to the lack of the appropriate anatomical information.

The anaesthetised sheep was injected with 257 MBq of ^18^F-FDG and scanned 30 min post-injection for 30 min. During the scan, the breathing of the animal was mechanically controlled by a ventilating machine. The cardiac and respiratory traces were recorded during the PET scan, using an ECG tracking device and a respiratory belt (AZ-733V, ANZAI Medical Co.). Triggers corresponding to the peak R-wave of the ECG and to the peak-inspiration of the breathing signal were inserted into the PET list-mode by these external tracking devices. They were then exploited to perform off-line phase-based gating of the PET dataset using in-house developed software (five respiratory gates and ten cardiac gates). After the gating, the list-mode corresponding to end-expiration and end-diastole was reconstructed using OSEM3D, OSEM3D+RR and the TV prior (w = 0.005).

A qualitative comparison of the images at the lesion sites was performed.

## Results

### Choice of the reconstruction parameters

We made use of the combined information from the bias-noise and contrast-noise plots, alongside with the line profiles through the middle slice of the heart and the opinion of an experienced nuclear medicine clinician, to define which of the Bowsher reconstructions was best to proceed with for the full subsequent analysis of the recovery and visibility of the simulated lesions.

**Fig. 6:**
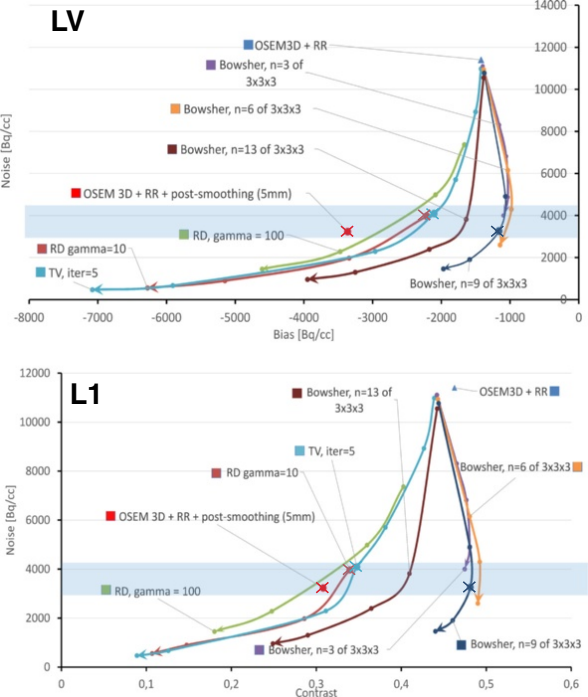
Bias-noise plot for the LV (*top pane*) and contrast-noise plot for the non-transmural lesion L1 (*bottom pane*). When the Bowsher prior is indicated, the MR is used as anatomical information during reconstruction. Twenty noise realizations per reconstruction were used. The *light-blue stripe* represents the noise range designated as clinically acceptable

Figure 6 shows the bias-noise plot for the LV and the contrast-noise curve for L1, for the different reconstruction algorithms and parameter choices. The curves are produced by varying the weight of the priors. Initially, by visually analysing the same reconstructions at different noise levels, we empirically selected those corresponding to a noise level of 3000–4500 (highlighted in blue in Fig. 6). The bias- and contrast-noise plots confirmed that this was an acceptable compromise between the noise and the bias levels, both of which should be as low as possible. Most of the curves in the plots bend towards worse contrast/bias values when going below this noise level. In other words, by further lowering the noise, the LV bias would become excessive, and the lesion contrast would start to decrease. We verified that these conclusions were in agreement with the opinion of our nuclear medicine expert.

**Fig. 7:**
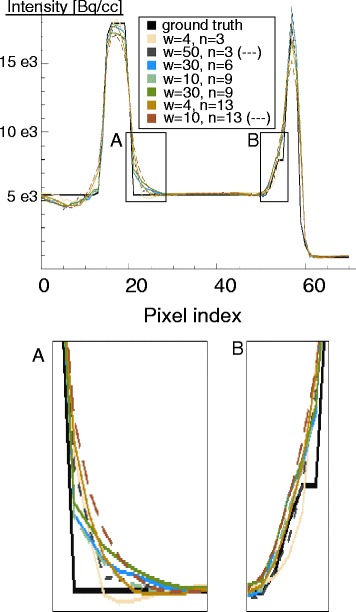
Profiles through the middle slice of the heart. Noise-free reconstructions

To supplement the results of the bias-noise and contrast-noise analysis, the profiles through the middle slice of the heart (Fig. 7) were also considered. The Bowsher reconstructions with 13 neighbours and high weights were discarded, as they appear artificially smooth and they fail to correctly restore both the activity peaks and the sharp intensity transitions. The reconstructions with three neighbours show the best edge preservation, but the noise pattern they display looks unnatural especially at higher weights. The reconstructions with six and nine neighbours appeared the best for the structures of interest, featuring a less artificial look and yet fairly good preservation of the edges. After a visual inspection of the reconstructed images, performed by an experienced nuclear medicine physician, a weight of 10 and a *n*=9 was chosen for the Bowsher reconstructions. For the TV prior, a weight of 0.005 was chosen to match the selected noise level. For the same reason, the paramenters chosen for the RD prior were *β*=4 and *γ*=10. The markers of the final selected reconstructions are in bold and crossed in the bias-noise and contrast-noise plots (Fig. 6).

### Visual analysis and septum-lateral profiles

**Fig. 8:**
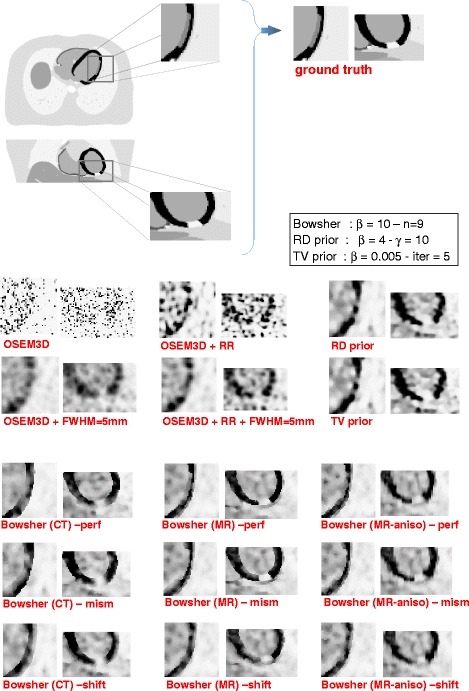
Zoomed in images of the lesions reconstructed using the different reconstruction algorithms (example of 1 noise realization). 3i42s + 2i24s + 2i1s iteration scheme. The RD and the TV priors represent a good choice in case no anatomical information is available, if the anatomical information does not highlight the lesions or if the anatomical information is mismatched (see Bowsher (HRCT) or Bowsher (MR)-shift). When the anatomical image is matching the PET dataset *and* it clearly differentiates between healthy and scarred tissue, then it is definitely the preferred choice (Bowsher(MR)-perf). Please note that the OSEM3D reconstructions suffer more than others from severe noise; this is due to the long iteration scheme, applied to ensure convergence and consistency with the other reconstructions. The suffix *-shift* indicates the case where the anatomy is displaced by 2 mm in the x and z direction, while *-mism* represents the case where the heart in the anatomical information has the same morphology but it is in a slightly different cardiac phase relative to the PET dataset

As Fig. 8 shows the HRCT-based prior moderately outperformed the non-anatomy-based priors in terms of contrast recovery of the ventricles and volume delineation, provided that the anatomical information was well aligned to the corresponding PET image. There was no improvement in terms of lesion visualisation, due to the fact that the HRCT does not show any intensity difference between lesions and healthy tissue. The edge-preserving priors, on the other hand, produced visually appealing images, with better contrast-to-noise ratios than the current standard for PET reconstruction (OSEM3D+RR+post-smoothing). The use of MR as anatomical information led to better delineation of the lesions. If, however, the anatomical information was shifted or mismatched when compared to the activity image, the delineation of the LV and of the lesions was affected and artefactual hypo-perfused areas appear in the LV. The use of anatomical information with non-ideal resolution (i.e. anisotropic MR) slightly affected the image quality. However, in most cases, the anisotropic resolution of the MR was not the bottleneck for the reconstruction with anatomical information.

**Fig. 9:**
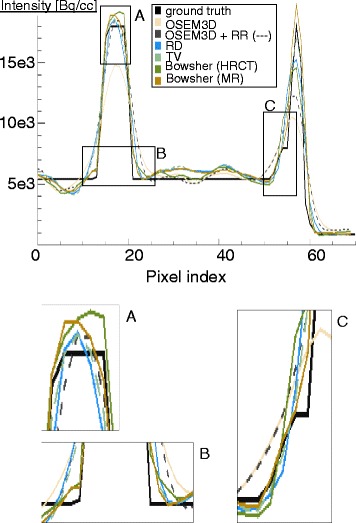
Profiles of the LV activity computed through the middle plane of the heart. The OSEM3D, OSEM3D+RR (both with a 5-mm post-smoothing), the two edge-preserving priors (TV with *w*=0.005, RD with *w*=4 and *γ*= 10) and the two anatomy-based priors (both with *w*=10 and *n*=9) are compared. These profiles are the mean over 20 noise realizations. In the bottom part, a zoomed-in version of the most interesting areas is shown. *a* and *b* demonstrate that a similar behaviour can be expected between MR-based and CT-based anatomical information, where no lesions are considered. In pane *c*, the MR reconstruction is the one that best follows the contours of the lesion

The profiles computed through the middle plane of the heart (Fig. 9) confirmed these observations. If perfectly aligned to the PET dataset, both HRCT and MR performed equally well as anatomical priors (leftmost part of the profile), except when the lesion was considered (rightmost part). There, again, the CT-based anatomical prior suffered from the fact that no information on the lesion was present, and therefore blurred the activity of the LV over it. The MR-based prior, on the contrary, managed to correctly recover the activity. The use of MRs that were not perfectly aligned to the PET dataset was not particularly deleterious for the mid-ventricular profile (not shown).

### Figures of merit

**Fig. 10:**
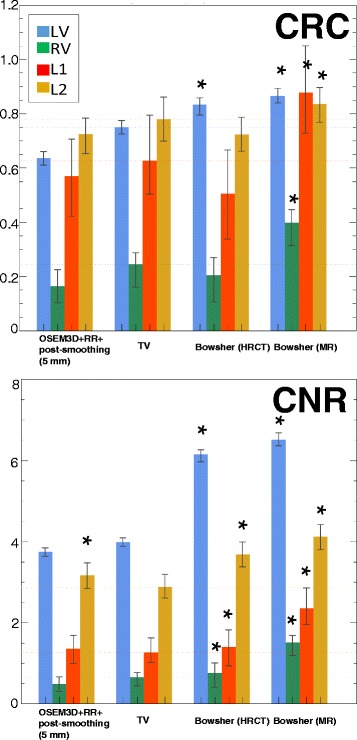
CRC and CNR comparison, edge-preserving prior (TV) vs ideal anatomical priors, calculated on 25 noise realizations. Mean values and min/max error bars. *Significantly better than TV. TV was chosen as reference as it yielded the *overall* best RC, CRC and CNR in comparison to all other non-anatomy-based reconstructions

The CRC and CNR were calculated for all the selected reconstructions. As a first step, we compared the results obtained with the current standard for reconstruction (OSEM3D+RR+post-smoothing) to the images obtained with the edge-preserving, non-anatomical prior (TV) and the two types of anatomical prior (perfectly matching HRCT and MR, respectively). Figure 10 describes the findings. When the lesion is not visible in the anatomy (i.e. HRCT), the use of anatomical priors does not improve the contrast recovery of the lesions, when compared to results obtained with the non-anatomical priors. However, the superior noise performance of the anatomy-based prior makes the lesions slightly better visible in such reconstructions (Fig. 10, Bowsher(HRCT) columns, CRC vs CNR). The same Figure and the results of the *t* test indicate that the improvement of the CNR is moderate (but significant) for Bowsher(HRCT) compared to TV, particularly when a non-transmural lesion (red column) is concerned. When the lesion is visible in the anatomy (i.e., MR), the anatomical prior significantly improves both contrast recovery and noise properties of all the anatomies.

**Fig. 11:**
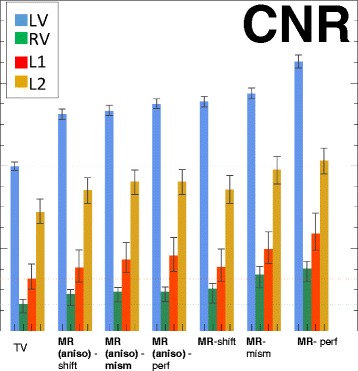
CNR comparison, edge-preserving prior (TV) vs non-ideal anatomical priors, calculated on 25 noise realizations. Mean values and min/max error bars. All reconstructions are significantly better than the edge-preserving prior (TV). The suffix *-shift* indicates the case where the MR is shifted by 2 mm in the x and z direction, whereas *-mism* indicates the case where the MR is in a slightly different cardiac phase relative to the PET)

Secondly, we further analysed the behaviour of the best anatomy-based reconstruction when the conditions are not ideal, i.e. the alignment between the anatomical and the PET dataset is disrupted or the anatomical information is acquired with anisotropic resolution (Fig. 11). The use of a shifted or mismatched anatomy affects both quantification and detectability, with more errors caused by rigid shift than cardiac phase mismatch. The use of a shifted or mismatched anatomy also introduced small, artefactually hypo-perfused areas (not shown), which could possibly be interpreted as false positives. The use of anatomical information with non-ideal resolution (i.e. anisotropic MR) does affect CRC and CNR. However, in most cases it still outperforms the non-anatomy-based priors.

**Fig. 12:**
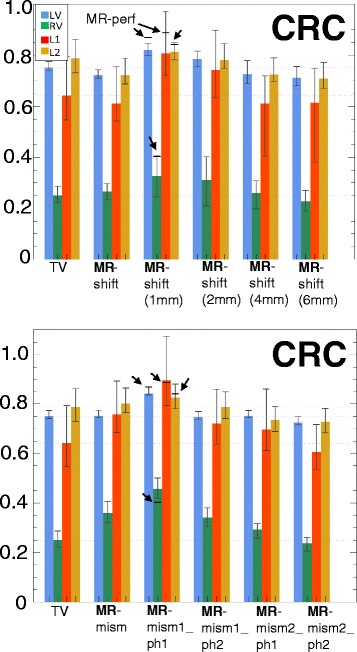
CRC comparison with different shifts or mismatches. Contrast recovery coefficients (CRC) for the different cardiac regions when different shifts (*top*) or mismatches (*bottom*) are applied to the MR before using it as anatomical information during reconstruction. The values here presented are the mean, maximum and minimum values over ten shift directions per shift magnitude and over ten noise realizations per mismatched MR, respectively. The MR-mism and MR-shift reconstructions are the same as in Fig. 11 (MR-shift is shifted by 2 mm in the x and z direction, MR-mism is in a slightly different cardiac phase)

A comparison of all the CRCs (Fig. 12, top) and the CNRs (not shown) of the Bowsher(MR)-based reconstructions with varying shifts was also performed, to verify the robustness of the findings to different degrees of misalignment. If the overall shift in the three directions is small enough (≤2 mm), the contrast recovery of the lesions is still acceptable and improved when compared to images obtained using a non-anatomical prior during reconstruction. On the other hand, particular attention needs to be paid when the shifts are greater than 2 mm. In this case, both the CRC and the CNR of the lesions worsen, when compared to TV. Similar conclusions can be drawn for the different mismatches (Fig. 12, bottom graph). If the misalignment between the image used as anatomical information and the PET image is small enough (e.g. mism1-ph1), the good performances of the anatomical prior are maintained. The stronger the mismatches, the higher the decrease in both CRC and CNR, particularly those of the lesions.

**Fig. 13:**
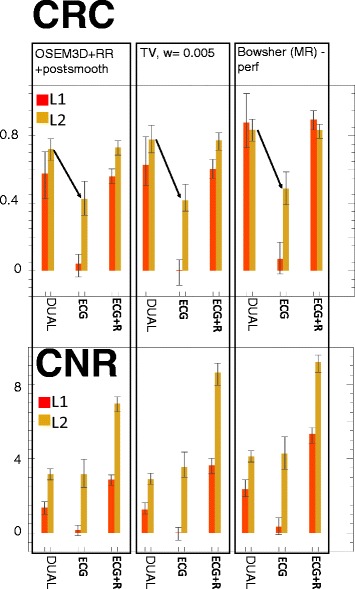
Effect of motion correction on CRC and CNR. The *top* plot shows the changes in contrast recovery when motion compensation is fully performed (*DUAL*, as in dual-gated studies), or only ECG gating is performed (*ECG*, one cardiac gate blurred by the respiratory motion) or if ECG gating is performed starting from a dataset where the respiratory motion has been compensated (*ECG+R*, one cardiac gate, less noisy)

The effect of motion correction is clear in Fig. 13. If the respiratory motion correction is neglected and only ECG gating is performed on the original list-mode dataset (*ECG* bars in the Figure), the lesions are hardly distinguishable due to the motion blur. On the other hand, the use of images with better noise properties and perfectly compensated for respiratory motion (*ECG+R* bars in the Figure) demonstrate that the better noise level improves the CNR as expected, and that the correct recovery of the contrast is independent of the noise level.

### Bull’s eye plots

The bull’s eye plots add insight into the activity distribution in both lesions. An in-depth analysis of the LV quantification is out of the scope of this work.

The transmural lesion is clearly identifiable in the bull’s eye plots of all reconstructions. The non-transmural lesion, on the other hand, should only be visible in the mean-count PMs, while it should be fully hidden in the max-count PMs, given the fact that such plots have been generated by taking the maximum intensity over the thickness of the LV.

**Fig. 14:**
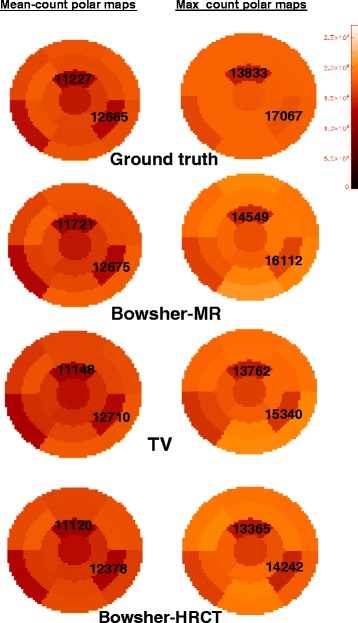
Mean-count vs max-count Bull’s eye plots. Segmental 17-segments polar maps obtained from the reconstructions of one noise realization. *Left column*: mean-count polar maps of a selection of the reconstructions performed. The Bowsher reconstruction with MR is overall the most similar to the ground truth, both in absolute and relative terms. In the *right column*, the max-count polar maps for the same reconstructions are presented. Again, the Bowsher reconstruction with MR produces the polar map with the best intensity preservation, when compared to all other reconstructions

The right column of Fig. 14 illustrates what is actually obtained. In the max-count polar maps, the healthy tissue situated close to the non-transmural lesion is often reconstructed with decreased intensity due to PVE or to incorrect PVC. Therefore, the bull’s eye plots will show an area of decreased activity, as if the segment contained a fully transmural lesion. In the ideal-case scenario, however, the better the healthy portion of the LV is reconstructed, the less we should notice the presence of the non-transmural lesion in the max-count PM. This is the case for the reconstructions with the MR used as anatomical prior, which is the one that most accurately reproduces the polar map of the ground truth.

On the other hand, this result, despite correct, might hide the presence of a non-transmural lesion to the clinician’s eye. In order to overcome this inconvenience and keep track of the non-transmural lesion in the bull’s eye plots, the use of a mean-count polar map is advised. The anatomical prior allows an accurate delineation of the LV, thus avoiding errors due to incorrect definition of the LV boundaries. At the same time, the presence of a hypo-perfused area is correctly revealed.

In both cases, the use of bull’s eye plots needs to be accompanied by a thorough observation of the corresponding short and long axis slices, especially if anatomy-based PVC is performed during reconstruction, in order to correctly differentiate between transmural and non-transmural lesions.

**Fig. 15:**
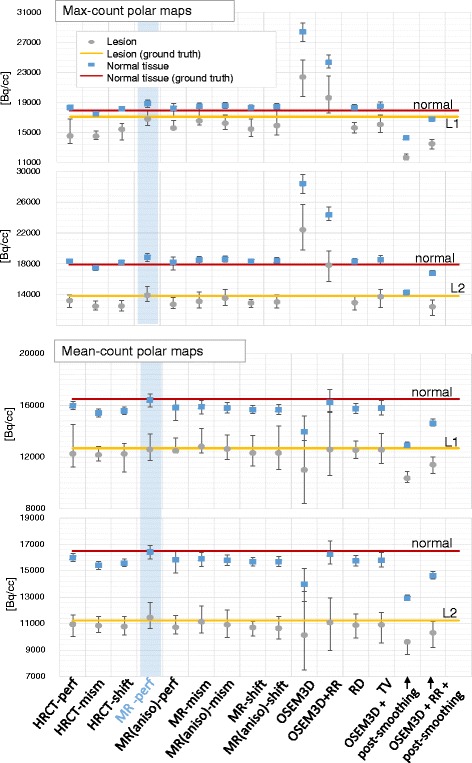
The mean, minimum and maximum values (over 25 noise realizations) of the normal and lesioned regions, computed on the corresponding segments of the polar maps, are compared. On the two graphs from the *top*, the values computed from the max-count polar maps are illustrated. On the two *bottom graphs*, the values computed from the mean-count polar maps are presented. For each type of polar map, the *top* parts (*first* and *third graph*) compare the intensity of the *normal region to* the intensity of the *non-transmural lesion (L1)*, using the different reconstruction algorithms, whereas the *bottom* parts (second and fourth graph) show the *same information for the transmural lesion (L2)*. The *solid lines* represent the mean values computed in the ground truth in the normal (*red*) and lesioned (*yellow*) tissues. The *light-blue stripe* highlights the reconstruction algorithm (MR-perf) that performs best in terms of similarity of the mean to the ground truth. To be noted that the mean-count polar maps of the OSEM3D+RR, despite being a good approximation of the ground truth *on average*, have very large error bars indicating that it can have very poor values for individual noise realisations

Figure 15 shows the mean values of the normal and lesioned regions, computed on the corresponding segments of the polar maps, for 25 noise realizations.

When the max-count polar maps are used and the non-transmural lesion is considered (top-left pane of Fig. 15), the mean intensity of the segment corresponding to the non-transmural lesion (L1) is, in the ground truth, very close to the mean intensity of the surrounding normal tissue. As expected, this behaviour is best reproduced when the Bowsher with a perfectly matching MR is used as anatomical information (light blue stripe). This is the reconstruction technique that gets closest to the segmental values of the L1 and L2 in ground truth, too. All other reconstruction algorithms artificially increase the contrast between the L1-segment and the normal tissue, due to the incorrect recovery of the surrounding healthy tissue (e.g., when the CT or the non-anatomical priors are used). The OSEM3D and OSEM3D+RR without smoothing are extremely noisy; hence, the mean segmental values of the max-count polar maps are all over-estimated. A post-smoothing applied to such reconstructions preserves the contrast between normal and lesioned segments (anyway higher than what it should be), and it underestimates the segmental value for the non-transmural lesion L1.

When the transmural lesion is considered (*bottom-left pane* of Fig. 15), all MAP reconstructions behave similarly and preserve both contrast and actual values. This is consistent with the fact that the maximum count over the thickness is anyway zero, regardless the alignment of the anatomical information or the prior used during reconstruction.

If a better representation of the ground truth values is aimed at, the use of mean-count polar maps is recommended. In this case, the mean intensity of the normal tissue decreases for almost all reconstruction algorithms except the Bowsher-MR and the OSEM3D with RR. In the case of OSEM3D+RR, the computation of a mean polar map (using the LV delineation obtained from the Bowsher(MR)-PET) does mitigate the sharp peaks of activity at the centre of the LV of the OSEM3D+RR reconstructions, which are the Gibbs artefacts due to the process of RR. This holds true as long as a correct delineation (in this case, the one from the MR-based reconstructions) is used. As far as the non-transmural lesion L1 is concerned (top-right pane of Fig. 15), the mean segmental values reflect the mean intensity of the ground truth when both a perfectly matching MR or edge-preserving priors are used. The use of a misaligned MR does hamper the quantification of both normal and lesioned regions, similarly to what the use of an HRCT does. The HRCT does not produce correct results due to the absence of pericardium and of the lesion in the anatomical image. The transmural lesion, again, suffers less from mismatches or incorrect lesion delineation in the anatomical image (bottom-right pane of Fig. 15).

These results are confirmed by the visual inspection of the polar maps. A selection of the bull’s eye plots compared to the ground truth is on Fig. 14.

### Proof of concept: animal dataset

**Fig. 16:**
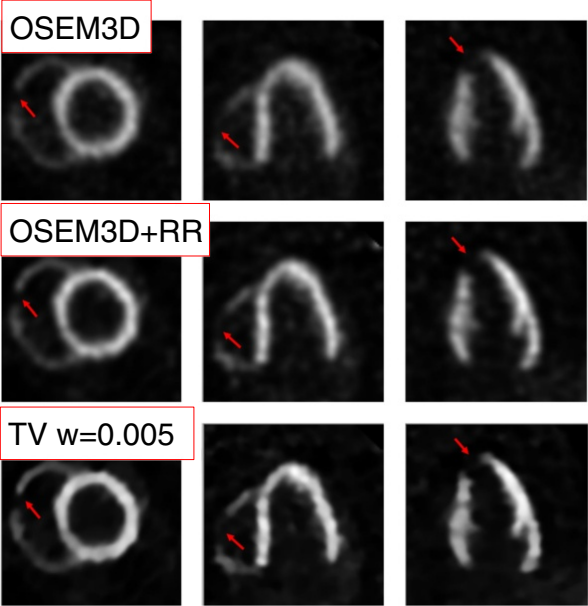
Animal dataset: a proof of concept. OSEM3D, OSEM3D+RR and TV reconstructions were retroactively performed on a measured animal dataset, after motion correction is applied. Both lesions are more clearly identifiable in the TV reconstruction. From left to right: short axis, horizontal and vertical long-axis views

The images resulting from the reconstruction of the animal dataset are in Fig. 16. The overall image quality improves when using the TV prior, with a noise-suppression and contrast-enhancement effect that is similar to the one observed in the simulation study. The boundaries of both lesions are also more clearly defined when using TV.

## Discussion

In the first place, it is important to emphasize the reason why a single cardiac and respiratory PET frame, obtained by double gating the original dataset, was considered here for PVC instead of a motion-corrected PET dataset (using all available data). Several methods to obtain motion fields with which the PET dataset could be corrected for motion have been proposed in the past: using a dynamic CT acquisition [30], via estimation of the motion fields directly from the PET dataset [5, 6] or using motion fields derived from a truly simultaneous PET/MR acquisition [7]. In the case of a shorter scan, for example, using a single cardiac gate from a study with motion correction would increase the fraction of the counts that are used to create the motion-free image. However, the additional radiation burden associated with the CT acquisition, the possibly inaccurate and non-trivial motion estimation from the PET dataset and the non-availability of truly-simultaneous PET/MR devices in most of the PET centres, made us opt for simulating the worst-case scenario where a simple double-gating pass was applied to the PET dataset. In this way, all PET datasets are equally noisy and are equally unaffected by possibly inaccurate motion-correction issues, and a fair comparison of the MR-based and CT-based PVC could be performed. In addition, the results obtained in the simulated, worst-case scenario are also representative for the case where the cardiac uptake was limited, or if the PET scan time was further reduced to optimize the clinical workflow or to limit gross patient motion during the scan [31]. We believe that most of the findings of this work would also apply to reconstructed PET datasets with better statistics and/or accurately corrected for motion.

A second point of interest emerges from the analysis of the preparatory results of this study. The correct choice of the prior parameters (weight, amount of edge preservation, number of neighbours considered) is the first fundamental task that needs to be performed before any reconstruction-based PVC. Despite being cumbersome (multiple trial reconstructions are needed, in order to find the best parameters to be used), this step is crucial for obtaining images that represent an acceptable compromise between intensity bias and noise suppression, and, most importantly, that are clinically acceptable.

Once the optimal parameters for reconstruction have been found, PVC of doubly-gated cardiac PET images with anatomical information is a delicate task that requires careful evaluation. The use of anatomical information that does not highlight scar tissue (e.g. HRCT or MR reconstructions where the lesion-to-LV contrast is absent) is not worth the effort and the extra radiation burden for the patient, particularly for lesion detection. If its alignment to the PET dataset was perfect, it would effectively suppress noise and make transmural lesions clearer, provided that the weight of the prior information was not too strong and the appropriate number of neighbours was chosen for the reconstruction. The visibility of the non-transmural lesions would be only marginally improved. If only anatomical information that does *not* highlight the scars can be made available, the improvement in lesion contrast and recovery would be so marginal that its acquisition would not be worth the effort. A non-anatomical, edge-preserving prior applied during reconstruction would be the best alternative.

If the anatomical image does differentiate between normal and scar tissue (e.g. via a properly chosen MR sequence), then its use is advisable for both absolute quantification and improved lesion detectability. However, it is of extreme importance to accurately align (≤2 mm mismatch) the anatomical and activity images before proceeding with PVC. If such alignment is not fully achieved, the risk of artefacts in the reconstructions is high and might compromise the diagnostic accuracy. The anisotropic resolution of the anatomical information decreases the performances of the MR-based PVC, particularly if the structures of interest are below the spatial resolution of the anatomical information. However, the anisotropy of the MR does not seem to represent a bottleneck for PVC with anatomical information, as long as a correct alignment is achieved.

The analysis of the polar plots suggests that any of the evaluated MAP algorithms would produce a fairly good preservation of lesion-to-normal tissue contrast. The use of misaligned or shifted anatomical information is not harmful if polar maps are used, particularly if the mismatches are reasonably small. In fact, even if the absolute quantification of the segments might be affected by the use of the wrong anatomy, the contrast between the lesioned segments and the normal segments is not degraded. Therefore, for the sole purpose of identification of segments with lesions in the Bull’s eye plots of the LV, the use of any of these MAP algorithms would be acceptable but the anatomical information would not introduce any significant improvement in lesion detection. The polar maps also confirm what was previously noticed in the reconstructed images. If the anatomical information is used, the absolute quantification of all the segments improves when compared to all other algorithms. However, to achieve such improvements, the anatomical image (MR) needs to be perfectly aligned to the PET dataset. In case the alignment is disrupted, the performances of the anatomy-based reconstructions are comparable to those of the reconstructions simply enhanced with an edge-preserving prior.

This simulation study analyses the effects of a badly aligned anatomical image relative to the PET dataset for the purpose of PVC of lesions in PET cardiac datasets. A few limitations of this study come from the ideal-case scenario we are currently considering. First of all, we utilize a single cardiac and respiratory PET gate, assuming that a perfect motion correction of the PET dataset is performed. We believe that this is the best way to clearly assess the effect of the different partial volume correction techniques, without confounding the effect of the motion blur with the partial volume effect. This said, we acknowledge that the breathing and beating motion are a tangible and cumbersome issue when cardiac datasets are of interest and that in the current clinical practice, there is often no time nor means to perform a fully dual-gated PET reconstruction. For this reason, we performed an additional simulation study where we purposely did not correct for the respiratory motion (i.e. only ECG gating is performed, which can be easily done in clinical practice) and we just applied the PVC techniques on such datasets. With this additional study, we demonstrated that the heart motions (both due to breathing and beating) need to be corrected for in order for PVC to successfully take place. The optimal compensation of one of the two motions would improve the noise properties of the resulting reconstructions, in all cases. However, a further improvement in lesion visibility can always be observed when the prior information is used. In addition, even in the best-case scenario where dual gating can be performed, sometimes a perfect motion correction simply cannot be achieved. This can be due to delays or imperfections in the insertion of the triggers in the PET dataset, or to gross patient motion that can occur during the scan and is not corrected for, or to the violated assumption of correlation between the external motion tracked by the external tracking devices (e.g. respiratory belts) and the internal motion of the heart, or to the unpredictable motion pattern of a real, patient heart (e.g. deformations occurring during the breathing), or to the too simplistic gating method used for motion correction. In all these non-ideal situations, the gating process can only be correct to a certain extent. If the heart does not behave as modelled by the gating software, part of the activity will be misplaced, and any of the PVC techniques presented would simply smooth over activity regions regardless of how well motion correction was performed.

We believe that the PVC should to be performed only when motion, randoms, scatter and attenuation correction are performed in the most accurate possible way. If this was not the case, the activity distribution before PVC would not be correct in the first place, and hence, any PVC technique would obviously not improve our knowledge on the actual, underlying activity distribution (on the contrary, it would even possibly emphasize areas of wrongly placed activity).

In this regard, it is important to discuss the need for random and scatter correction. Both corrections are necessary for a correct and artefact-free estimation of the myocardial distribution of activity. In our case, we simplify the scenario by assuming the absence of random and scatter from our simulated datasets. The results here presented would change, in the direction of a worsening of the image bias, if these corrections were not correctly applied. An analysis of the effects of a wrong random and scatter correction was out of the scope of this study.

It is also necessary to underline that this study uses a perfectly matching attenuation image to correct for attenuation during the PET measurement. In a previous study [10], we proved that the use of a mismatched attenuation image can affect the subsequent PVC of the PET dataset with anatomical information in two ways. Firstly, the use of a mismatched attenuation correction can create fake, hypo-active areas in the LV depending on the direction and the amount of the displacement of the AC CT relative to the PET dataset. This could result by itself in errors in the quantification of the (lesions in the) LV. In addition, the correction of the PET dataset with a mismatched attenuation image can mislead the registration of the anatomical image for PVC to the activity image. This, in turn, would result in wrong PVC (as in one of our simulated cases where the anatomy is mismatched relative to the activity image). Therefore, the results presented in this work have to be considered as the best-case scenario, where the attenuation map used does not hamper the registration nor the quantification and image quality.

Investigating ways to improve the alignment between the PET and the anatomical dataset was out of the scope of this work. In the cases where the PET and the anatomical images are slightly misaligned, a registration of the anatomical information to the PET datasets might mitigate the issue, but the registration process is not straightforward. Rigid-registration algorithms normally have an accuracy of 3–4 mm if inter-modality registration is performed [32]. However, we here observed that these mismatch values are not acceptable if the PET dataset needs to be reconstructed with anatomical information. The use of non-rigid registration to correct for small deformations, despite appealing, appears difficult due to the often high level of noise in the PET dataset. One possible solution would be to correct the PET dataset with an edge-preserving prior first, then align it to the anatomical information image of choice and, once a correct alignment is achieved, proceed with a new reconstruction of the PET dataset with the help of anatomical information. We briefly investigated this alternative, but the first results obtained did not represent an improvement to the reconstructions with the original misalignment (not shown). A more in-depth investigation will be needed, to find the optimal registration algorithm and parameters that could lead to a more encouraging result in this direction.

Finally, we would like to conclude with a comment on the feasibility of the acquisition of gated anatomical information of sufficient quality for PVC. As for the acquisition of an HRCT, while hybrid PET/CT devices do not have the necessary resolution for obtaining CT images for PVC, dedicated CT scanners with high spatial and temporal resolution are nowadays part of the clinical practice. With these scanners, it is possible to obtain a ‘frozen’ image of the heart in a specific cardiac and respiratory phase, with a very low radiation burden for the patient [33]. When the patient requirements asked by the developers of such scanners are met (e.g. the heartbeat of the patient has to be below 70 bpm for optimal image quality), the images obtained with these dedicated CT devices are suited for PVC of healthy (non-lesioned) cardiac tissue. However, the usage of separate or dedicated HRCT scanners for the acquisition of anatomical images for cardiac PVC seems impractical for several reasons. Firstly, in a HRCT, the lesions are not well visible, thus limiting the applicability of the PVC method using HRCT to a better recovery of healthy tissue. Secondly, the fact of moving the patient from one scanner (the PET/CT) to the other (HRCT) for the acquisition of the anatomical information could increase the chances of small motions and deformations of the heart between the two scans, which would in turn increase the chances of incorrect PVC. This would be an issue both for the use of a dedicated HRCT and MR scanner. Additionally, for what dedicated MRI scanners are concerned, the acquisition of images with sufficient temporal and spatial resolution for PVC would be problematic, due to the limited allocated time for such scans. For all these reasons, great interest goes in the recent development of integrated, truly simultaneous PET/MR devices, which we believe could foster the application of anatomy-based PVC techniques in the clinical practice. The use of these scanners would allow the acquisition of an MR image of sufficient resolution, as its acquisition could be performed simultaneously with the PET scan (and, therefore, last at least several minutes). Moreover, the problem of moving the patient from one scanner to the other (or, even worse, to scan the patient on two different days, for the PET and the MR scan separately) would be fully avoided. Navigators, self-navigated methods and acceleration methods for obtaining MR images with a good temporal resolution, accurately corrected for the periodic cardiac motion and with less motion artefacts (e.g. ghosting) are successfully being used nowadays and are under continuous improvement [34, 35]. We believe that the ongoing research in the field of simultaneous PET/MR (which includes solving the problem of attenuation correction of the PET datasets, using only the MR or the PET information [36]) would not only be useful for improved diagnosis, but it could be the only clinically feasible way to apply anatomy-based PVC on cardiac (and potentially other non-brain) PET datasets.

## Conclusions

The aim of this work was to assess the performances of edge-preserving priors for the purpose of lesion detection in cardiac ^18^F-FDG PET, in comparison with anatomy-based priors. We also aimed at highlighting the possible risks of anatomy-based PVC on lesion detection and quantification, which occur when the anatomical image is mismatched or shifted when compared to the corresponding PET dataset, and at comparing MR-based and CT-based PVC on lesion quantification.

This study concludes that, as far as lesions are concerned, the use of anatomical information in the form of HRCT is not worth the acquisition and use, as it only marginally improves the noise properties of the images at the price of reduced contrast recovery of the lesions and of an increased radiation burden for the patient. The quantitative evaluations (bull’s eye plots, CRC, RC, profiles) show that its performances are equal or inferior to a well-tuned edge-preserving prior. If an MR image acquired with a sequence that well differentiates between the various tissue types is available, and its alignment to a previously reconstructed PET image is optimal (≤2 mm), the recovery and visibility of the lesions does improve when a new, anatomy-based PET reconstruction using such well-aligned anatomical information is performed. However, great care needs to be taken in choosing the correct set of parameters for reconstruction and in verifying the correct alignment of the two datasets. If the correct alignment between the anatomical and the PET dataset is in doubt and cannot be otherwise improved, it is recommended to proceed with a non-anatomical prior (e.g. TV) applied during reconstruction. This is also recommended when only the bull’s eye plots are of interest.
